# Approaches to Cognitive Modeling in Dynamic Systems Control

**DOI:** 10.3389/fpsyg.2017.02032

**Published:** 2017-11-29

**Authors:** Daniel V. Holt, Magda Osman

**Affiliations:** ^1^Department of Psychology, Heidelberg University, Heidelberg, Germany; ^2^Department of Biological and Experimental Psychology, School of Biological and Chemical Sciences, Queen Mary University of London, London, United Kingdom

**Keywords:** dynamic decision making, complex problem solving, cognitive modeling, instance-based learning, heuristics, causal learning

## Abstract

Much of human decision making occurs in dynamic situations where decision makers have to control a number of interrelated elements (dynamic systems control). Although in recent years progress has been made toward assessing individual differences in control performance, the cognitive processes underlying exploration and control of dynamic systems are not yet well understood. In this perspectives article we examine the contribution of different approaches to modeling cognition in dynamic systems control, including instance-based learning, heuristic models, complex knowledge-based models and models of causal learning. We conclude that each approach has particular strengths in modeling certain aspects of cognition in dynamic systems control. In particular, Bayesian models of causal learning and hybrid models combining heuristic strategies with reinforcement learning appear to be promising avenues for further work in this field.

## Introduction

Handling dynamic systems is a common requirement in everyday life, ranging from operating a novel technical device, to managing a company or understanding the dynamics of social relationships. Considerable progress has been made toward assessing dynamic systems control (DSC) as a cognitive skill, particularly in educational contexts (OECD, 2014; Schoppek and Fischer, 2015). However, the development of cognitive theories which describe and explain the mental processes underlying this skill seems to lag behind. In this perspectives article we therefore examine what computational models of cognition can contribute toward an improved understanding of different cognitive processes involved in DSC. For this purpose, we briefly review several approaches to cognitive modeling in DSC, summarize their relative strengths and weaknesses, and conclude with what we perceive as promising routes for future research.

Dynamic systems control can be defined as a form of dynamic decision making that requires (1) a series of interrelated decisions (2) in interaction with a dynamic system inducing states of subjective uncertainty (3) with the aim of attaining (and maintaining) a goal state and/or to explore the system and possible courses of action (also see Edwards, 1962; Osman, 2010). Subjective uncertainty may be caused by random fluctuations of the system but also by limited knowledge of the system’s structure and its dynamics (Osman, 2010). In cognitive research DSC is typically investigated using computer-simulated *microworlds*, which are the focus of the present paper (but see Klein et al., 1993, for a different approach). Microworlds emulate cognitively relevant features of DSC situations (e.g., limited information, delayed feedback, time pressure) framed in a semantically plausible setting such as managing a company or fighting a forest fire (Brehmer and Dörner, 1993; Gonzalez et al., 2005).

In this article, the terms *cognitive model* or *computational model* (*of cognition*) refer to any mechanistic account of cognitive processes that is sufficiently specified to allow a computer-based implementation yielding quantitative predictions of behavior, cognitive processing steps, or neural activity (Lewandowsky and Farrell, 2011). Computational models enforce conceptual completeness, as all functionally relevant properties of a theory have to be made explicit for its computational implementation. Furthermore, computational models generate precise predictions of data patterns that can be empirically tested, which most verbally expressed theories are unable to do with the same level of precision. Models implemented as computer programs can also be used for simulation-based exploration to investigate how varying parameter settings, assumptions about cognitive processes, or simulated task demands affect model behavior. For a comprehensive treatment of computational modeling in cognition - including its challenges and problems - please refer to Lewandowsky and Farrell (2011).

We will now consider the contribution of different approaches to modeling cognition in DSC with selected examples. Our brief review begins with comparatively simple and knowledge-lean modeling paradigms, moving toward approaches involving more complex strategies and knowledge structures (see **Table 1** for an overview).

**Table 1:** Basic approaches to cognitive modeling in dynamic systems control (DSC).

Approach	Requirements	Strengths	Limitations	Examples
*Instance-based learning*: Acquisition of situation-response associations guided by outcome feedback.	Frequent exposure to similar states of the DSC task. Prior knowledge can be minimal.	Simple formalism Universal applicability across different domains High neural and cognitive plausibility	No representation of causal knowledge Requires direct outcome feedback	Taatgen and Wallach, 2002; Gonzalez et al., 2003
*Heuristic models*: Simple “rules of thumb” with low cognitive requirements.	Situations for which control heuristics based on prior knowledge are available.	Structurally simple models Role of heuristics in decision making well supported Can be extended with reinforcement learning	No representation of causal knowledge Need to establish suitable heuristics for each domain	Reichert and Dörner, 1988; Sterman, 1989
*Complex knowledge-based models*: Complex cognitive strategies and abstract mental representations.	Typically requires considerable task-specific prior knowledge.	Modeling of complex knowledge structures and reasoning strategies	Can be very complex Often highly task-specific, limited transfer	Schunn and Anderson, 1998; Schoppek, 2002
*Causal learning*: Bayesian induction of causal relations from observing system behavior.	Task sufficiently simple to allow causal attribution. Prior knowledge can be minimal.	Comprehensive formalism for representing causal knowledge, uncertainty and knowledge updating	Have not yet been directly applied to system control tasks	Steyvers et al., 2003; Meder et al., 2010

## Instance-Based Learning

Instance-based learning (IBL) models have arguably been among the most successful approaches to cognitive modeling in DSC. They are based on a simple principle analogous to reinforcement learning: control actions that lead to a successful outcome become reinforced in memory and will therefore more likely be remembered and enacted when a similar situation is encountered in the future (Gonzalez et al., 2003). The application of this learning principle in DSC can be traced back to Berry and Broadbent’s (1984) semi work on knowledge acquisition in dynamic systems. In their deceptively simple task, the production of a simulated sugar factory had to be kept in a target range by adjusting the number of workers. Surprisingly, most participants were unable to verbally describe the system’s structure despite being able to control it above chance level. As an explanation, Broadbent et al. (1986) suggested that people might store instances of situation-action combinations they have experienced in memory (e.g., hiring X workers when the current number of workers is Y and the current production is Z). Subsequent decision making in turn is based on retrieving instance memories similar to the situation encountered. Those instances repeatedly associated with successful outcomes (e.g., reaching the target production) become reinforced in memory and gradually start to dominate behavior, although no verbalizable representation of the system’s structure has been formed.

A computational IBL model of the sugar factory task has been implemented by Taatgen and Wallach (2002; also see Dienes and Fahey, 1995) using the ACT-R cognitive architecture (Anderson et al., 2004). Each instance was modeled as a unit in declarative memory encoding current state, action and outcome. On encountering a given system state, the model searches for instance memories that are similar to the current state and have led to the target outcome in the past, taking into account how often the instance memory has been retrieved before. The model requires only two production rules and closely fits human behavior. A model of the sugar factory relying on a similar associative learning mechanism was implemented by Gibson et al. (1997) using an artificial neural network. This illustrates that the basic learning principle is independent of any specific modeling architecture. IBL has also been applied to modeling more complex tasks such as controlling an array of pumps in a simulated water purification plant where the system state changes in real time (Gonzalez et al., 2003). The model included a blending mechanism to interpolate information across related instances and relied on a simple decision heuristic as fallback when instance memories were insufficient. A generic implementation of the IBL framework has been made available by Dutt and Gonzalez (2012) to make IBL modeling accessible to non-expert modelers.

An approach similar to IBL was used by Glass and Osman (2017; also Osman et al., 2015) to model learning in a simple dynamic system with continuous input and output variables. Instead of relying on a cognitive architecture, the authors adapted a general-purpose reinforcement learning algorithm to this task (Sutton and Barto, 1998). The model updated the reinforcement history of input variables after each trial depending on how much the last action reduced goal distance. This results in model behavior broadly similar to IBL. Glass and Osman (2017) specifically focused on modeling group differences between young and old adults in terms of exploration vs. exploitation behavior. They mapped this behavioral preference on the noise parameter affecting the choice of input values. Reinforcement learning has also been used to model conflicts between short- and long-term goals and how unreliable information affects learning in dynamic control (Gureckis and Love, 2009).

In sum, IBL and reinforcement learning models have been successfully used to explain different aspects of exploration and control in DSC. The basic mechanism is simple, cognitively plausible and requires only few task-specific assumptions. However, IBL models critically depend on the availability of immediate outcome feedback and the frequent repetition of similar decision situations to facilitate learning, which limits the type of task they can be applied to (see **Table 1**). Furthermore, IBL models cannot easily explain how people acquire explicit knowledge of the causal structure of a system, which is a central element of some DSC tasks (e.g., Kluge, 2008; Wüstenberg et al., 2012).

## Heuristic Models

Heuristics-based approaches to DSC assume that people rely on simple rule-of-thumb-type decision strategies for controlling dynamic systems. These strategies do not guarantee an optimal result, but allow to achieve reasonable outcomes across a range of conditions with limited cognitive effort (Brehmer, 2005; Shah and Oppenheimer, 2008). Characteristically, heuristic strategies do neither involve complex reasoning nor a detailed mental representation of the problem structure. Empirical research has shown that heuristics can explain adaptive behavior in many decision making situations as well as common errors and biases (Gilovich et al., 2002; Gigerenzer and Brighton, 2009). Furthermore, due to their simplicity heuristics are relatively easy to implement as cognitive models (e.g., Marewski and Mehlhorn, 2011).

The use of heuristics has also been proposed as an explanation for decision making behavior in DSC (e.g., Brehmer and Elg, 2005; Cronin et al., 2009). One of the best known examples of a computational heuristic model in DSC is Sterman’s (1989) model of decision making in a supply chain management task. The model is based on the anchoring-and-adjustment heuristic (Tversky and Kahneman, 1974), which involves substituting an unknown quantity (supplies ordered) with a related known quantity (sales forecast), adjusting for further influences (current stock level). Data simulated using this heuristic closely match human behavior and reproduce the characteristic oscillation between over- and undersupply that arises from ignoring system delays (Sterman, 1989). The cold store temperature regulation task (Reichert and Dörner, 1988) poses a similar challenge to participants, as the system responds with delay to changed inputs. Reichert and Dörner’s (1988) model captures how participants gradually learn to control the system by applying incremental changes to a proportional-control heuristic after unsuccessful control attempts. A conceptually related adaptive heuristic strategy is directional learning: if increasing an input improves the outcome then continue to increase it, otherwise decrease it. Computational models of directional learning have for example been used to model behavior in dynamic economic games, such as the multiple-round prisoner’s dilemma or the ultimatum game (Selten and Stoecker, 1986; Grosskopf, 2003).

Heuristic models can also be combined with reinforcement learning to simulate how people learn to choose among competing heuristic strategies. The probability of selecting a strategy depends on the outcomes that this strategy has produced in the past (Erev and Barron, 2005). For example, Gonzalez et al. (2009) used this approach to model response times in a dynamic radar detection and decision making task. The model fitted human data about as well as an alternative IBL model, although it transferred less well to changed task conditions. In contrast, Fum and Stocco (2003) found that a strategy-based learning model of the sugar factory task performed better under changed conditions than the corresponding IBL model of Taatgen and Wallach (2002). It appears that the transfer across situations depends on the details of the task, the type of training, and the strategies implemented. Strategy-based learning can also be applied to highly complex tasks such as fighting a simulated forest fire (De Obeso Orendain and Wood, 2012). In this model four high-level heuristic strategies competed (e.g., dropping water on the fire or creating a barrier to contain the fire), which were modeled in great detail. The model successfully reproduced how varying the conditions in the training phase affected preferences for particular strategies in later transfer.

In sum, there are several good examples of heuristics-based and hybrid models that address pertinent theoretical questions in DSC (e.g., handling delays, transfer of learning). Although incorporating more task-specific knowledge than pure learning models, models of this type typically still have a relatively simple basic structure. When heuristic models are extended with more complex strategies and abstract knowledge structures they gradually transform into complex knowledge-based models, which we will consider next.

## Complex Knowledge-Based Models

Complex knowledge-based models involve the creation and transformation of abstract knowledge structures combined with complex cognitive strategies. This corresponds to the notion of mental models guiding reasoning and actions in DSC (e.g., Brehmer, 1992). Models of this type are often implemented as production systems, i.e., sets of if-then-rules that act on knowledge objects or initiate external behaviors (Newell and Simon, 1972). This flexible mechanism allows to express a wide range of strategies up to expert performance at the expense of resulting in very complex and task-specific models in many cases.

Anzai (1984) presented one of the first models of this type in the context of navigating a simulated large ship which responded with considerable delay. Based on the analysis of verbal protocols, Anzai (1984) designed a production system model that qualitatively captured the acquisition of control knowledge in novices and experts. Later studies showed how production system approaches can be extended to model performance even in complex real-time decision making tasks such as that of a radar operator or flying a commercial jet plane (Schoelles and Gray, 2000; Schoppek and Boehm-Davis, 2004). In modeling real-time control tasks the emphasis usually lies on modeling the time course of control performance and typical errors.

A different class of DSC task that requires scientific reasoning to explore the causal structure of unknown systems has been particularly relevant in the recent educationally oriented wave of DSC research (Herde et al., 2016). Schoppek (2002) proposed a fine-grained cognitive model that was able to systematically explore and control a small dynamic system based on linear equations. The model incorporated an explicit mental representation of the system’s structure and mental calculation steps to derive input values. This strategy is sufficiently general to control any simple dynamic system based on linear equations (Funke, 2001). The model was able to simulate the effects of different degrees of system knowledge and strategic sophistication and compared favorably to results from human data (Schoppek, 2002). Similarly, Schunn and Anderson (1998) applied a production system approach to model the task of designing scientific experiments (given a restricted set of design options) and drawing conclusions about causal relations from the simulated results. This model was able to successfully capture performance differences between experts and novices by modeling their respective domain knowledge and exploration strategies.

An apparent advantage of complex knowledge-based models is their ability to explain how causal system knowledge combined with reasoning strategies informs the actions that people take. It seems difficult to imagine how some forms of DSC could be explained without recourse to reasoning and abstract knowledge representation, for example, extrapolating system behavior in new situations or hypothesis testing and rule-deduction in discovery learning. However, models of this type are often neither simple nor elegant and require the inclusion of considerable task-specific knowledge (Taatgen and Anderson, 2010).

## Bayesian CaUsaL Learning

Another modeling approach with a focus on structural knowledge is the use of Bayesian networks to model causal learning (Meder et al., 2010; Osman, 2017). Bayesian networks represent a formalism to express the strength of belief in causal hypotheses and provide a principled mechanism based on Bayesian inference for updating beliefs as new evidence becomes available (Holyoak and Cheng, 2011). For instance, Steyvers et al. (2003) used Bayesian networks to model human causal learning either by passive observation of a causal system or through direct interaction with it. This addresses a central aspect of DSC, system exploration and the formation of structural knowledge, although the approach has yet to be applied to DSC tasks requiring goal-directed control.

From a DSC perspective, the strength of Bayesian models of causal learning lies in the nuanced representation of causal structures and probabilistic dependencies combined with a mechanism for updating this knowledge from experience. This makes them a strong contender for explaining structural knowledge acquisition in DSC tasks with an exploration focus (e.g., Kluge, 2008; Wüstenberg et al., 2012). A Bayesian approach provides a formal account of the causal environment from which it is possible to deduce a suitable course of action, given the state of knowledge (including the level of uncertainty) a person has of the world at that time (Osman, 2010, 2017).

## Synthesis and Concluding Remarks

In pursuit of answering the question what cognitive modeling can contribute to DSC research we have considered several approaches (see **Table 1**). In terms of knowledge-lean modeling approaches, IBL strikes a good balance between simplicity, cognitive plausibility and explanatory power for a range of DSC tasks. On the downside, IBL has strict task requirements (e.g., availability of feedback, repeated decisions) and cannot easily explain the acquisition of causal knowledge or extrapolation to unfamiliar conditions. Heuristic models have no universal task requirements and can be combined with learning mechanisms to achieve a similar adaptivity as IBL models. However, since effective heuristics rely on exploiting the structure of the environment, finding suitable candidate heuristics for a given task can be a considerable challenge and any specific heuristics-based model is only applicable to a particular niche (Marewski and Schooler, 2011).

Complex knowledge-based models are probably the most domain-specific type of model. They require strong assumptions about knowledge structures and cognitive procedures used by decision makers. If this information is available, it is possible to model skilled expert performance, elaborate reasoning strategies, and the acquisition of explicit structural knowledge, e.g., through active hypothesis testing. With respect to modeling causal knowledge, Bayesian models of causal learning provide an interesting alternative.

They offer an integrated account for representing and updating causal knowledge in a coherent framework, including the representation of epistemic uncertainty. However, these models have so far not been directly applied to model control in microworld DSC tasks.

As our discussion shows, each modeling approach has its particular strengths and weaknesses, which render it suitable for particular modeling tasks. For researchers it therefore seems important to select a modeling approach that matches the research question and that suits the task to be modeled. For example, modeling the process of acquiring explicit causal knowledge in simple dynamic systems through hypothesis testing (e.g., Wüstenberg et al., 2012) naturally maps on complex knowledge-based models or Bayesian models of causal learning but is likely to run into difficulties when approached with an IBL framework.

In general, we think that the rapidly advancing theories in causal learning and hybrid models combining heuristic strategies with reinforcement learning offer considerable untapped potential for cognitive modeling in DSC. Causal learning directly addresses the core issue of DSC tasks focusing on causal exploration (e.g., Steyvers et al., 2003). However, in order to simulate full task performance models of this type would need to be extended by including interaction with the task. Hybrid models in turn may be most suitable to model behavior in complex decision making tasks (e.g., Danner et al., 2011), where (heuristically guided) information reduction and gradual strategy adaptation are central for task performance.

We furthermore propose that computational models based on cognitively plausible process assumptions (e.g., reinforcement learning, use of simple heuristics, Bayesian knowledge updating) could be used as a yardstick for evaluating human performance in DSC (see Brehmer, 2005). This stands in contrast to using mathematical optimization or optimal rational strategies as a benchmark for performance (e.g., Sager et al., 2011). Defining rationally optimal strategies in DSC does have its place, for example when designing decision support systems. However, from a behavioral perspective the question of “what is *maximally* possible” is often less relevant than “what is *humanly* possible,” given the realities of incomplete information and limited cognitive capacity (Klein, 2002).

In conclusion, computational models of cognition appear to offer a promising path for advancing research and theory development in DSC. Computational approaches have successfully been used to model a range of cognitive phenomena in different domains of DSC. Promising starting points for further developments include, for example, recent advances in causal learning and hybrid models which combine simple heuristics with reinforcement learning mechanisms. Computational modeling of cognitive processes in DSC remains a constructive challenge that probes – and ideally enhances – our understanding of human behavior in complex dynamic environments.

## Author Contributions

Both authors contributed to writing the manuscript and approved it for publication.

## Conflict of Interest Statement

The authors declare that the research was conducted in the absence of any commercial or financial relationships that could be construed as a potential conflict of interest.

## References

[B1] Anderson J. R. Bothell D. Byrne M. D. Douglass S. Lebiere C. Qin Y. (2004). An integrated theory of the mind. *Psychol. Rev.* 111:1036. 10.1037/0033-295X.111.4.1036 15482072

[B2] Anzai Y. (1984). Cognitive control of real-time event-driven systems. *Cogn. Sci.* 8 221–254. 10.1016/S0364-0213(84)80002-6

[B3] Berry D. C. Broadbent D. E. (1984). On the relationship between task performance and associated verbalizable knowledge. *Q. J. Exp. Psychol.* 36 209–231. 10.1080/14640748408402156

[B4] Brehmer B. (1992). Dynamic decision making: human control of complex systems. *Acta Psychol.* 81 211–241. 10.1016/0001-6918(92)90019-A 1462786

[B5] Brehmer B. (2005). Micro-worlds and the circular relation between people and their environment. *Theor. Issues Ergon. Sci.* 6 73–93. 10.1080/14639220512331311580

[B6] Brehmer B. Dörner D. (1993). Experiments with computer-simulated microworlds: escaping both the narrow straits of the laboratory and the deep blue sea of the field study. *Comput. Hum. Behav.* 9 171–184. 10.1016/0747-5632(93)90005-D

[B7] Brehmer B. Elg F. (2005). “Heuristics in dynamic decision making: coping with the time constants of a dynamic task by doing something else,” in *Proceedings of the 23rd International Conference of the System Dynamics Society*, eds Sterman J. D. Repenning N. P. Langer R. S. Rowe J. I. Yanni J. M. Boston, MA.

[B8] Broadbent D. E. FitzGerald P. Broadbent M. H. (1986). Implicit and explicit knowledge in the control of complex systems. *Br. J. Psychol.* 77 33–50. 10.1111/j.2044-8295.1986.tb01979.x

[B9] Cronin M. A. Gonzalez C. Sterman J. D. (2009). Why don’t well-educated adults understand accumulation? A challenge to researchers, educators, and citizens. *Organ. Behav. Hum. Decis. Process.* 108 116–130. 10.1016/j.obhdp.2008.03.003

[B10] Danner D. Hagemann D. Holt D. V. Hager M. Schankin A. Wüstenberg S. (2011). Measuring performance in dynamic decision making: reliability and validity of the tailorshop simulation. *J. Individ. Differ.* 32 225–233. 10.1027/1614-0001/a000055

[B11] De Obeso Orendain A. D. O. Wood S. (2012). “An account of cognitive flexibility and inflexibility for a complex dynamic task,” in *Proceedings of the 11th International Conference on Cognitive Modeling*, eds Russwinkel N. Drewitz U. Rijn H. van (Berlin: TU Berlin), 49–54.

[B12] Dienes Z. Fahey R. (1995). Role of specific instances in controlling a dynamic system. *J. Exp. Psychol.* 21:848 10.1037/0278-7393.21.4.848 9745379

[B13] Dutt V. Gonzalez C. (2012). Making instance-based learning theory usable and understandable: the instance-based learning tool. *Comput. Hum. Behav.* 28 1227–1240. 10.1016/j.chb.2012.02.006

[B14] Edwards W. (1962). Dynamic decision theory and probabilistic information processing. *Hum. Factors* 4 59–74. 10.1177/001872086200400201 25741290

[B15] Erev I. Barron G. (2005). On adaptation, maximization, and reinforcement learning among cognitive strategies. *Psychol. Rev.* 112:912. 10.1037/0033-295X.112.4.912 16262473

[B16] Funke J. (2001). Dynamic systems as tools for analysing human judgement. *Think. Reason.* 7 69–89. 10.1080/13546780042000046

[B17] Fum D. Stocco A. (2003). “Instance vs. rule-based learning in controlling a dynamic system,” in *Proceedings of the 5th International Conference on Cognitive Modelling*, eds Detje F. Dörner D. Schaub H. (Bamberg: Universitäts-Verlag), 105–110.

[B18] Gibson F. P. Fichman M. Plaut D. C. (1997). Learning in dynamic decision tasks: computational model and empirical evidence. *Organ. Behav. Hum. Decis. Process.* 71 1–35. 10.1006/obhd.1997.2712

[B19] Gigerenzer G. Brighton H. (2009). Homo heuristicus: why biased minds make better inferences. *Top. Cogn. Sci.* 1 107–143. 10.1111/j.1756-8765.2008.01006.x 25164802

[B20] Gilovich T. Griffin D. Kahneman D. (eds) (2002). *Heuristics and Biases: The Psychology of Intuitive Judgment.* Cambridge: Cambridge University Press 10.1017/CBO9780511808098

[B21] Glass B. D. Osman M. (2017). Positive explorers: modeling dynamic control in normal aging. *Aging Neuropsychol. Cogn.* 24 62–79. 10.1080/13825585.2016.1171290 27141924

[B22] Gonzalez C. Dutt V. Healy A. F. Young M. D. Bourne L. E. Jr. (2009). “Comparison of instance and strategy models in ACT-R,” in *Proceedings of the 9th International Conference on Cognitive Modeling*, eds Peebles H. D. Cooper R. (Manchester: University of Manchester).

[B23] Gonzalez C. Lerch J. F. Lebiere C. (2003). Instance-based learning in dynamic decision making. *Cogn. Sci.* 27 591–635. 10.1016/S0364-0213(03)00031-4

[B24] Gonzalez C. Vanyukov P. Martin M. K. (2005). The use of microworlds to study dynamic decision making. *Comput. Hum. Behav.* 21 273–286. 10.1016/j.chb.2004.02.014

[B25] Grosskopf B. (2003). Reinforcement and directional learning in the ultimatum game with responder competition. *Exp. Econ.* 6 141–158. 10.1023/A:10253050

[B26] Gureckis T. M. Love B. C. (2009). Learning in noise: dynamic decision-making in a variable environment. *J. Math. Psychol.* 53 180–193. 10.1016/j.jmp.2009.02.004 20161328 PMC2678746

[B27] Herde C. N. Wüstenberg S. Greiff S. (2016). Assessment of complex problem solving: what we know and what we don’t know. *Appl. Meas. Educ.* 29 265–277. 10.1080/08957347.2016.1209208

[B28] Holyoak K. J. Cheng P. W. (2011). Causal learning and inference as a rational process: the new synthesis. *Annu. Rev. Psychol.* 62 135–163. 10.1146/annurev.psych.121208.131634 21126179

[B29] Klein G. (2002). “The fiction of optimization,” in *Bounded Rationality: The Adaptive Toolbox*, eds Gigerenzer G. Selten R. (Cambridge, MA: MIT Press).

[B30] Klein G. A. Orasanu J. Calderwood R. Zsambok C. E. (1993). *Decision Making in Action: Models and Methods.* Norwood, NJ: Ablex.

[B31] Kluge A. (2008). Performance assessments with microworlds and their difficulty. *Appl. Psychol. Meas.* 32 156–180. 10.1177/0146621607300015 8741982

[B32] Lewandowsky S. Farrell S. (2011). *Computational Modeling in Cognition: Principles and Practice.* Thousand Oaks, CA: Sage 10.4135/9781483349428

[B33] Marewski J. N. Mehlhorn K. (2011). Using the ACT-R architecture to specify 39 quantitative process models of decision making. *Judgm. Decis. Mak.* 6 439–519.

[B34] Marewski J. N. Schooler L. J. (2011). Cognitive niches: an ecological model of strategy selection. *Psychol. Rev.* 118 393–437. 10.1037/a0024143 21744978

[B35] Meder B. Gerstenberg T. Hagmayer Y. Waldmann M. R. (2010). Observing and intervening: rational and heuristic models of causal decision making. *Open Psychol. J.* 3 119–135. 10.2174/1874350101003010119

[B36] Newell A. Simon H. A. (1972). *Human Problem Solving.* Englewood Cliffs, NJ: Prentice-Hall.

[B37] OECD (2014). *PISA 2012 Results Creative Problem Solving: Students’ Skills in Tackling Real-life Problems*, Vol. V Paris: OECD 10.1787/9789264208070-en

[B38] Osman M. (2010). Controlling uncertainty: a review of human behavior in complex dynamic environments. *Psychol. Bull.* 136 65–86. 10.1037/a0017815 20063926

[B39] Osman M. (2017). “Planning and Control,” in *Oxford Handbook of Causal Reasoning*, ed. Waldmann M. (Oxford: Oxford University Press).

[B40] Osman M. Glass B. D. Hola Z. (2015). Approaches to learning to control dynamic uncertainty. *Systems* 3 211–236. 10.3390/systems3040211 28880183

[B41] Reichert U. Dörner D. (1988). Heurismen beim Umgang mit einem “einfachen” dynamischem system [heuristics in handling a “simple” dynamic system]. *Spr. Kogn.* 7 12–24.

[B42] Sager S. Barth C. M. Diedam H. Engelhart M. Funke J. (2011). Optimization as an analysis tool for human complex problem solving. *SIAM J. Optim.* 21 936–959. 10.1137/11082018X 26858747

[B43] Schoelles M. J. Gray W. D. (2000). “Argus prime: modeling emergent microstrategies in a complex simulated task environment,” in *Proceedings of the 3rd International Conference on Cognitive Modeling*, eds Taatgen N. Aasman J. (Veenendaal: Universal Press), 260–270.

[B44] Schoppek W. (2002). Examples, rules, and strategies in the control of dynamic systems. *Cogn. Sci. Q.* 2 63–92.

[B45] Schoppek W. Boehm-Davis D. A. (2004). Opportunities and challenges of modeling user behavior in complex real world tasks. *MMI Interaktiv* 7 47–60.

[B46] Schoppek W. Fischer A. (2015). Complex problem solving—single ability or complex phenomenon? *Front. Psychol.* 6:1669. 10.3389/fpsyg.2015.01669 26594184 PMC4633517

[B47] Schunn C. D. Anderson J. R. (1998). “Scientific discovery,” in *The Atomic Components of Thought*, eds Anderson J. R. Lebiere C. (New York, NY: Psychology Press), 385–427.

[B48] Selten R. Stoecker R. (1986). End behavior in sequences of finite Prisoner’s Dilemma supergames A learning theory approach. *J. Econ. Behav. Organ.* 7 47–70. 10.1016/0167-2681(86)90021-1

[B49] Shah A. K. Oppenheimer D. M. (2008). Heuristics made easy: an effort-reduction framework. *Psychol. Bull.* 134:207. 10.1037/0033-2909.134.2.207 18298269

[B50] Sterman J. D. (1989). Modeling managerial behavior: misperceptions of feedback in a dynamic decision making experiment. *Manag. Sci.* 35 321–339. 10.1287/mnsc.35.3.321

[B51] Steyvers M. Tenenbaum J. B. Wagenmakers E. J. Blum B. (2003). Inferring causal networks from observations and interventions. *Cogn. Sci.* 27 453–489. 10.1016/S0364-0213(03)00010-7

[B52] Sutton R. S. Barto A. G. (1998). *Reinforcement Learning: An Introduction.* Cambridge, MA: MIT Press.

[B53] Taatgen N. Anderson J. R. (2010). The past, present, and future of cognitive architectures. *Top. Cogn. Sci.* 2 693–704. 10.1111/j.1756-8765.2009.01063.x 25164050

[B54] Taatgen N. A. Wallach D. (2002). Whether skill acquisition is rule or instance based is determined by the structure of the task. *Cogn. Sci. Q.* 2 163–204.

[B55] Tversky A. Kahneman D. (1974). Judgment under uncertainty: heuristics and biases. *Science* 185 1124–1131. 10.1126/science.185.4157.1124 17835457

[B56] Wüstenberg S. Greiff S. Funke J. (2012). Complex problem solving—More than reasoning? *Intelligence* 40 1–14. 10.1016/j.intell.2011.11.003

